# Does interpregnancy BMI change affect the risk of complications in the second pregnancy? Analysis of pooled data from Aberdeen, Finland and Malta

**DOI:** 10.1038/s41366-021-00971-7

**Published:** 2021-10-04

**Authors:** Dylan Peter McClurg, Mika Gissler, Miriam Gatt, Jacqueline Wallace, Sohinee Bhattacharya

**Affiliations:** 1 Aberdeen Centre for Women’s Health Research, School of Medicine, Medical Sciences & Nutrition, University of Aberdeen, Aberdeen, UK; 2 THL Finnish Institute for Health and Welfare, Information Services Department, Helsinki, Finland; 3 Department of Molecular Medicine and Surgery, Karolinska Institute, Stockholm, Sweden; 4 Directorate for Health Information and Research, Strategy and Sustainability Division, Ministry for Health, Valletta, Malta; 5 Rowett Institute, School of Medicine, Medical Sciences and Nutrition, University of Aberdeen, Aberdeen, UK

**Keywords:** Medical research, Disease prevention, Weight management, Risk factors

## Abstract

**Objective:**

Weight management interventions during pregnancy have had limited success in reducing the risk of pregnancy complications. Focus has now shifted to pre-pregnancy counselling to optimise body weight before subsequent conception. We aimed to assess the effect of interpregnancy body mass index (BMI) change on the risk of perinatal complications in the second pregnancy.

**Methods:**

A cohort study was performed using pooled maternity data from Aberdeen, Finland and Malta. Women with a BMI change of ±2 kg/m^2^ between their first and second pregnancies were compared with those who were BMI stable (remained within ±2 kg/m^2^). Outcomes assessed included pre-eclampsia (PE), intrauterine growth restriction (IUGR), preterm birth, birth weight, and stillbirth in the second pregnancy. We also assessed the effect of unit change in BMI for PE and IUGR. Logistic regression was used to calculate odds ratios with 95% confidence intervals.

**Results:**

An increase of ≥2 kg/m^2^ between the first two pregnancies increased the risk of PE (1.66 (1.49–1.86)) and high birthweight (>4000 g) (1.06 (1.03–1.10)). A reduction of ≥2 kg/m^2^ increased the chance of IUGR (1.15 (1.01–1.31)) and preterm birth (1.14 (1.01–1.30)), while reducing the risk of instrumental delivery (0.75 (0.68–0.85)) and high birthweight (0.93 (0.87–0.98)). Reducing BMI did not significantly decrease PE risk in women with obesity or those with previous PE. A history of PE or IUGR in the first pregnancy was the strongest predictor of recurrence independent of interpregnancy BMI change (5.75 (5.30–6.24) and (7.44 (6.71–8.25), respectively).

**Conclusion:**

Changes in interpregnancy BMI have a modest impact on the risk of high birthweight, PE and IUGR in contrasting directions. However, a prior history of PE and IUGR is the dominant predictor of recurrence at second pregnancy.

## Introduction

The global pandemic of obesity and its myriad of complications has led to it becoming a major target for public health campaigns. Obesity during pregnancy represents an important preventable risk factor that is strongly associated with poorer maternal and foetal outcomes [1–5]. Unfortunately, interventions for weight loss initiated during pregnancy remain largely ineffective in reducing obstetric complications [6]. One possible explanation for this may be the short time interval for any significant physiological changes to take effect [7]. Alternatively, pre-pregnancy genetic or metabolic factors may also determine the occurrence of such complications and thus may not be particularly amenable to lifestyle alterations during pregnancy. As a result, women are now being targeted for interventions during the post-partum period in an attempt to prevent these complications from recurring. However, previous studies examining the effects of interpregnancy weight change and the risk of subsequent pregnancy complications are, on the whole, limited by their small sample sizes and/or recall bias from self-reported weight or body mass index (BMI) [8–16]. Furthermore, only a few have evaluated the impact of interpregnancy BMI change in high-risk patient groups, such as those with obesity or prior gestational complications [17–19]. Therefore, a better understanding of the relationship between interpregnancy weight change and adverse pregnancy outcomes is vital for developing evidence-based interventions and guidance, especially for those at high-risk of individual complications.

Avoiding the consequences of an unhealthy BMI by encouraging primigravid women to obtain a healthy pre-pregnancy weight is a high priority goal. Although a laudable aim, this is extremely difficult given that the majority of first pregnancies are unplanned, particularly in younger or socially deprived women [20]. As a result, preconception care alone is no longer tenable and more efforts, such as those targeting the interpregnancy period to achieve a healthy body weight prior to conceiving the subsequent pregnancy, may help reduce the overall risk of adverse perinatal outcomes.

Previous population-based studies have investigated interpregnancy BMI/weight change and its relationship with various pregnancy complications [11, 19, 21–28]. These studies have shown a strong association between interpregnancy weight change and the risk of premature delivery, pre-eclampsia (PE), extremes of placental weight, small or large for gestational age-births, and gestational diabetes [11, 19, 21–28]. One of the published large population-based cohort studies utilises data from the Swedish birth register (*n* = 151 025) [21] and demonstrates a linear relationship between interpregnancy weight gain and the first occurrence of PE during the second pregnancy. These results were replicated within a Scottish cohort where in addition to finding an increased risk of primary PE with interpregnancy weight gain specific to women who were overweight or had obesity at baseline [24], conversely demonstrated an increased risk of primary and recurrent SGA- birth with interpregnancy weight loss [19, 24]. As PE and IUGR or SGA frequently co-exist in pregnancies, it is imperative to understand the effects of interpregnancy weight change on PE and IUGR, especially in those who have previously experienced these serious complications. The aim of this study is to assess the effect of interpregnancy BMI change on the risk of perinatal complications including PE and IUGR recurrence in the second pregnancy.

## Methods

### Study design

We conducted a cohort study using routinely collected perinatal data from three countries: Scotland, Finland, and Malta. The population of interest were women with records of their first two consecutive singleton pregnancies, with no prior live or stillbirths, in the birth registries of the three countries. Our exposure variable, interpregnancy weight change, was estimated by calculating the difference between first and second pregnancy maternal BMI recorded at the time of the first antenatal visit (weight (kg)/height (m^2^)). Changes in BMI were categorised as; ≤2 kg/m^2^ (BMI reduced), −2 to 2 kg/m^2^ (BMI stable and reference group), and ≥2 kg/m^2^ (BMI increased). BMI changes of 2 kg/m^2^ were selected to maintain comparability with previous studies [19], and in consideration that a BMI change of 1 kg/m^2^ equates to only 2.6 kg (assuming an average height of 1.62 m) which may reflect natural weight fluctuations and not a true gain or loss of weight. The outcomes were any adverse perinatal events in the second pregnancy such as PE (hypertension with proteinuria during pregnancy), placenta praevia, placental abruption, IUGR (recorded as per ultrasound diagnosis), preterm birth (further grouped into delivery before 37/34/32 weeks of gestation), low birthweight (birth weight <2500 g), high birth weight (>4000 g), stillbirth, and mode of delivery (vaginal, instrumental, elective or emergency caesarean section (CS)). The following variables were considered as potential confounders: maternal age, BMI at first baseline pregnancy, socioeconomic status based on maternal occupation (Finland), paternal occupation (AMND) or maternal education (Malta), marital status, and smoking habits at the time of the second delivery. In addition, interpregnancy interval, year of delivery of the second baby, and the country of origin of the datasets were also tested for potential confounding effects. Maternal age was categorised into five categories as <20, 20–29, 30–35, 36–40 and ≥41 years. BMI was categorised as underweight (<18.5), normal weight (18.5–24.9), overweight (25–29.9), and obesity (≥30). Socioeconomic status was recoded into a binary variable: deprived (yes/no). Registrar General’s fathers’ occupation-based social class in the AMND was coded as ‘not deprived’ (I, II, IIIa) and the remainder as ‘deprived’. Data from Finland was coded ‘upper white-collar worker’ as ‘not deprived’, all others (lower white-collar, blue-collar and other including student and housewife) as ‘deprived’. Maternal education level in Malta was used as a social class with university-level education coded as ‘not deprived’ and below this level as ‘deprived’.

### Data sources

Data were obtained from three sources: the Aberdeen Maternity and Neonatal Databank (AMND) between 1986 and 2012 [29], the Finnish Medical Birth Register (MBR) between 2004 and 2014 [30], and the Maltese National Obstetric Information System (NOIS) between 1999 and 2015 [31]. Finnish MBR [30] and Maltese NOIS [31] are national perinatal registers that collect data on all maternities across the country while AMND collects data on all births within Aberdeen City District––a defined geographical region in the North East of Scotland [29]. De-identified data were extracted from the three sources and pooled to form a single database after standardising and recoding all key variables. From this database, women with information on their first two singleton births were selected for analysis and those with incomplete BMI records in either of their first two pregnancies were excluded.

### Statistical analysis

Descriptive statistics were presented as mean (SD) for normally distributed continuous variables and counts and proportions for categorical variables. Characteristics of the second pregnancy were compared between BMI stable and BMI changed cohorts using univariable analysis, chi-square test for categorical variables and ANOVA for continuous variables. A *p* value of ≤0.05 was considered statistically significant. Binary or multinomial logistic regression was used to calculate odds ratios (OR) with 95% confidence intervals (CI) for predefined perinatal outcomes of the second pregnancy for each of the exposed cohorts. Models were adjusted for potential confounders identified on univariable analysis plus the same adverse outcome in the previous pregnancy. Low and high birth weights were also adjusted for gestational age at delivery. We also examined weight change as a continuous variable and calculated the risk of PE and IUGR stratified by the BMI category in the first pregnancy. There were no data missing in the exposure and outcome variables due to the inclusion and exclusion criteria. Complete case analysis was performed with ≤5% of missing data in the covariates.

### Ethical permissions

Permission to analyse de-identified data were obtained from the Caldicott guardians of all three databases: the steering committee of the Aberdeen Maternity and Neonatal Databank (AMND 3/2016); Finnish Institute for Health and Welfare, Finland (THL 1719/5.05.00/2015); Directorate for Health Information Research Malta (28/04/2016). As routinely collected de-identified data were analysed, formal ethical approval was not considered necessary by the North of Scotland Research Ethics Service. This analysis was part of a collaborative project looking at the recurrence risk of stillbirth.

## Results

**Fig. 1:**
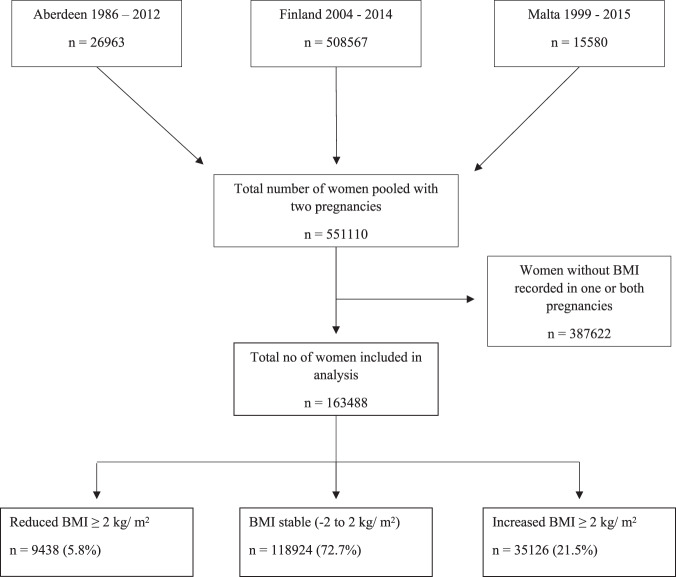
Flow chart. Flow chart demonstrating participant selection from respective databases.

After data cleaning and merging, there were 163 488 women with both first and second pregnancy records included in the analysis (Fig. 1). Table 1 presents the maternal characteristics at the time of the second pregnancy by BMI change. The timing of the first antenatal visit varied among the participants but 90% of women attended for their first antenatal visit before 12 weeks of gestation in both pregnancies. In the remaining 10% of late attendees, no association was found with maternal BMI or interpregnancy BMI change. Within the total cohort, 118,924 (72.7%) were BMI stable across the two pregnancies, while 9 438 (5.8%) had reduced, and 35 126 (21.5%) had increased their BMI by ≥2 kg/m^2^. Women who were aged <20 years were more likely to increase their BMI (0.7% versus 1.4%) while women who were aged 20 to 29 years were equally likely to increase or reduce their BMI (Table 1). Compared to BMI stable women, those who increased their BMI were more likely to fall into the overweight (18.5% versus 40.2%) or obesity (7.0% versus 33.4%) category at the time of their second pregnancy. On the other hand, women who reduced their BMI were found more frequently within the underweight (3.8% versus 5.4%), overweight (18.5% versus 22.7%), or obesity (7.0% versus 12.2%) category. Women exhibiting interpregnancy weight change were more likely to be socially deprived, smoke, and be single. Furthermore, women who had increased their BMI were more likely to have longer interpregnancy intervals (Table 1).

**Table 1:** Characteristics of second pregnancy in relation to BMI change.

Characteristics	BMI stable	Reduced BMI	Increased BMI	*p* value*
	*n* = 118,924 (72.7%)	*n* = 9 438 (5.8%)	*n* = 35 126 (21.5%)	
Maternal age (years)	29.82 (4.62)	28.92 (4.78)	28.99 (4.99)	**<0.001**
<20	777 (0.7%)	99 (1.0%)	481 (1.4%)	
20–29	54,761 (46.0%)	5150 (54.6%)	18,701 (53.2%)	
30–35	45,001 (37.8%)	2983 (31.6%)	10,925 (31.1%)	
36–40	16,129 (13.6%)	1057 (11.2%)	4349 (12.4%)	
≥41	2255 (1.9%)	148(1.6%)	670 (1.9%)	
BMI (kg/m^2^)	23.44 (4.02)	24.27 (4.08)	28.67 (5.35)	**<0.001**
Underweight	4465 (3.8%)	509 (5.4%)	26 (0.1%)	
Healthy weight	84,169 (70.8%)	5,626 (59.6%)	9266 (26.4%)	
Overweight	22,011 (18.5%)	2,147 (22.7%)	14,106 (40.2%)	
Obesity	8,279 (7.0%)	1,156 (12.2%)	11,728 (33.4%)	
Social class				**<0.001**
Not deprived	26,757 (22.9%)	1500 (16.2%)	5201 (15.2%)	
Deprived	87,789 (75.3%)	7261 (78.6%)	28,048 (82.1%)	
Missing	2116 (1.8%)	474 (5.1%)	935 (2.7%)	
Smoking				**<0.001**
Non-smoker	10,8231 (91.0%)	8055 (85.3%)	30,673 (87.3%)	
Smoker	8647 (7.3%)	1239 (13.1%)	3828 (10.9%)	
Missing	2046 (1.7%)	144 (1.5%)	625 (1.8%)	
Marital status				**<0.001**
Married/cohabiting	1,12,238 (94.4%)	8596 (91.1%)	32274 (91.9%)	
Single	6532 (5.5%)	821 (8.7%)	2782 (7.9%)	
Unknown	140 (0.1%)	20 (0.2%)	60 (0.2%)	
Interpregnancy Interval				**<0.001**
<2 years	66,153 (55.6%)	4899 (51.9%)	726 (7.7%)	
2–5 years	47,335 (39.8%)	3813 (40.4%)	14,328 (40.8%)	
>5 years	5434 (4.6%)	726 (7.7%)	3689 (10.5%)	
Year of delivery				**<0.001**
1986–1995	4989 (4.2%)	239 (2.5%)	1176 (3.3%)	
1996–2005	8134 (6.8%)	789 (8.4%)	2917 (8.3%)	
2006–2015	1,05,799 (89.0%)	8410 (89.1%)	31,032 (88.3%)	
Country of origin				**<0.001**
Aberdeen	14,538 (12.2%)	1021 (10.8%)	4616 (13.1%)	
Finland	98,795 (83.1%)	7354 (77.9%)	27,940 (79.5%)	
Malta	5591 (4.7%)	1063 (11.3%)	2570 (7.3%)	

*Statistically significant *p* values (<0.001) are shown as bold.

Table 2 presents the counts, proportions, and unadjusted and adjusted ORs (95% CIs) of outcomes of the second pregnancy in relation to interpregnancy BMI change. In comparison to weight stable BMI women (3.0%), both a reduction (3.6%) and an increase in BMI (5.1%) was associated with an enhanced incidence of PE (Table 2). Reduced BMI increased the rate of IUGR (2.7% versus 2.2% in weight stable women) while an increase in BMI was associated with a reduction (1.7%). Reduced BMI was also associated with a slight increase in the risk of preterm birth before 37 weeks (2.7% versus 3.1%) and 32 weeks (0.6% versus 0.8%). Women who had reduced their BMI were more likely to be delivered by elective or emergency CS but less likely to have an instrumental delivery. Women who had increased their BMI were also more likely to be delivered by both elective and emergency CS than their BMI stable counterparts (Table 2).

**Table 2:** Comparison of outcomes of second pregnancy among BMI stable (reference group, OR = 1), reduced and increased interpregnancy BMI.

Outcomes of 2nd pregnancy	BMI stable	Reduced BMI			Increased BMI		
	*n* = 118,924 (72.7%)	*n* = 9,438 (5.8%)			*n* = 35,126 (21.5%)		
	*n* (%)	*n* (%)	Unadjusted	Adjusted	*n* (%)	Unadjusted OR (95% CI)	Adjusted OR^a^ (95% CI)
			OR (95% CI)	OR (95% CI)			
PE	3,519 (3.0%)	340 (3.6%)	**1.23 (1.09–1.37)**	1.12 (0.99–1.26)	1,786 (5.1%)	**1.76 (1.66–1.86)**	**1.66 (1.49–1.86)**
Placenta praevia	726 (0.6%)	66 (0.7%)	1.15 (0.89–1.48)	1.22 (0.95–1.58)	206 (0.6%)	0.96 (0.82–1.12)	1.09 (0.91–1.30)
Placental abruption	336 (0.3%)	35 (0.4%)	1.31(0.93–1.86)	1.33 (0.93–1.91)	100 (0.3%)	1.01 (0.81–1.26)	0.91 (0.70–1.18)
IUGR	2,607 (2.2%)	254 (2.7%)	**1.24 (1.09–1.41)**	**1.15 (1.01–1.31)**	601 (1.7%)	**0.78 (0.71–0.85)**	0.94 (0.85–1.04)
Preterm birth							
<37 weeks	3,229 (2.7%)	297 (3.1%)	**1.16 (1.03–1.32)**	**1.14 (1.01–1.30)**	1,025 (2.9%)	0.93 (0.86–1.00)	0.96 (0.88–1.04)
<34 weeks	693 (0.6%)	71 (0.8%)	**1.30 (1.02–1.66)**	**1.33 (1.04–1.72)**	210 (0.6%)	0.97 (0.83–1.14)	0.99 (0.83–1.18)
<32 weeks	262 (0.2%)	27 (0.3%)	1.30 (0.88–1.96)	1.15 (0.75–1.75)	89 (0.3%)	0.87 (0.68–1.10)	0.90 (0.68–1.18)
Birthweight							
Low <2500 g	2,700 (2.3%)	248 (2.6%)	**1.16 (1.02–1.33)**	1.12 (0.97–1.28)	791 (2.3%)	0.99 (0.92–1.07)	1.05 (0.96–1.16)
High ≥4000 g	21,094 (17.7%)	1,619 (17.2%)	0.96 (0.91–1.02)	**0.93 (0.87–0.98)**	8,004 (22.8%)	**1.37 (1.33–1.41)**	**1.06 (1.03–1.10)**
Stillbirth	227 (0.2%)	20 (0.2%)	1.11 (0.70–1.76)	1.13 (0.70–1.81)	88 (0.3%)	1.31 (0.93–1.68)	1.14 (0.85–1.53)
Mode of delivery							
Instrumental	6,029 (5.1%)	365 (3.9%)	**0.78 (0.69–0.86)**	**0.75** (**0.68–0.85)**	1,746 (5.0%)	0.97 (0.91–1.02)	0.96 (0.90–1.03)
Elective CS	8,276 (7.0%)	825 (8.7%)	**1.29 (1.18–1.37)**	0.96 (0.88–1.05)	3,267 (9.3%)	0.71 (0.68–0.74)	0.96 (0.91–1.01)
Emergency CS	7,454 (6.3%)	657 (7.0%)	**1.12 (1.04–1.22)**	1.03 (0.94–1.13)	2,962 (8.4%)	0.70 (0.67–0.74)	0.97 (0.92–1.02)

Statistically significant odds ratios (*p* < 0.001) are shown as bold.

^a^All odds ratios are adjusted for maternal age, BMI, smoking, social class, marital status, interpregnancy interval, country of origin, year of delivery at the second pregnancy. In addition, models were adjusted for the same outcome in the previous pregnancy. Low and high birthweight was also adjusted for gestational age at delivery preterm birth was additionally adjusted for induction of labour.

After adjusting for potential confounding factors, a reduction of ≥2 kg/m^2^ was associated with an increased risk of IUGR (1.15 (1.01–1.31)), preterm birth before 37 weeks (1.14 (1.01–1.30)) as well as 34 weeks (1.33 (1.04–1.72)), and a reduced chance of instrumental delivery (0.75 (0.68–0.85)) (Table 2). Although the risk of PE was associated with a ≥2 kg/m^2^ decrease on unadjusted analysis, this was no longer statistically significant after adjustment (1.12 (0.99–1.26)) (Table 2). On the other hand, a BMI increase of ≥2 kg/m^2^ conferred an increased risk of PE 1.66 (1.49–1.86)), and high birthweight (1.06 (1.03–1.10)) in the second pregnancy (Table 2).

Table 3 presents the fully adjusted models in relation to two key clinical outcomes, PE and IUGR, with ORs for all predictor variables.

**Table 3:** Fully adjusted models for PE and IUGR in the second pregnancy.

Variables	PE aOR (95% CI)	IUGR aOR (95% CI)
	(*n* = 5645)	(*n* = 3462)
BMI change		
BMI reduced	1.12 (0.99–1.26)	**1.15 (1.01–1.31)**
BMI increased	**1.66 (1.49–1.86)**	0.94 (0.85–1.04)
Maternal age	**1.04 (1.03–1.05)**	**0.97 (0.96–0.98)**
BMI in 2nd pregnancy	**1.10 (1.09–1.11)**	**0.95 (0.94–0.95)**
Smoking	0.98 (0.95–1.00)	**1.05 (1.02–1.07)**
Marital status (single)	0.98 (0.91–1.06)	**1.11 (1.05–1.18)**
Deprived social class	**1.04 (1.02–1.05)**	**1.07 (1.04–1.10)**
Interpregnancy interval	**1.10 (1.08–1.12)**	**1.07 (1.05–1.10)**
Country of origin	0.92 (0.85–1.01)	**1.18 (1.04–1.33)**
History of same complication	**5.75 (5.30–6.24)**	**7.44 (6.71–8.25)**
Year of birth	**0.94 (0.93–0.95)**	**1.03 (1.02–1.04)**

Statistically significant odds ratios (*p* < 0.001) are shown as bold.

As previously shown in Table 2, women who increased their interpregnancy BMI by ≥2 kg/m^2^ were found to have an increased risk of PE on adjusted analyses (1.66 (1.49–1.86)) (Table 3). A prior history of PE was the strongest predictor of PE in the second pregnancy (5.75 (5.30–6.24)). In addition, increasing maternal age, (1.04 (1.03–1.05)), BMI status at the start of the second pregnancy (1.10 (1.09–1.11)), deprived social class (1.04 (1.02–1.05)), and an increased interpregnancy interval (1.10 (1.08–1.12)), were significantly associated with the occurrence of PE in the second pregnancy (Table 3).

Similar to PE, a previous history of IUGR was the greatest predictor of IUGR incidence in the second pregnancy (7.44 (6.71–8.25)) (Table 3). Furthermore, an interpregnancy BMI reduction (1.15 (1.01–1.31)), a younger maternal age 0.97 (0.96–0.98), a lower BMI at the start of the second pregnancy (1.15 (1.01–1.31)), smoking (1.05 (1.02 – 1.07)), single marital status (1.11 (1.05–1.18)), deprived social class (1.07 (1.04–1.10)), and increasing interpregnancy interval (1.07 (1.05– 1.10)) demonstrated significant associations of IUGR development in the second gestation (Table 3).

**Fig. 2:**
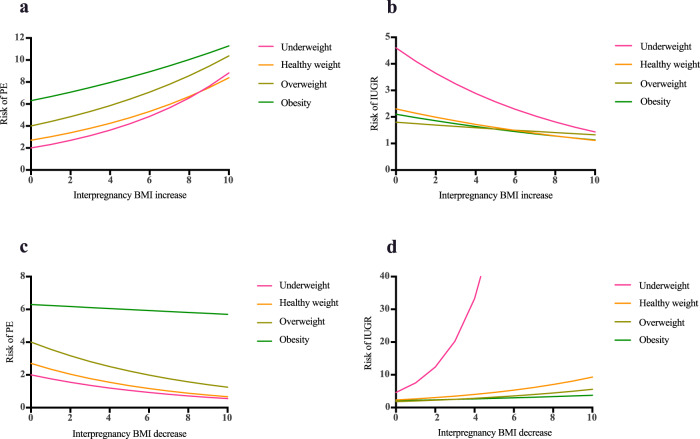
Interpregnancy BMI Unit change by BMI categories and risk of PE and IUGR. **a**, **b** Change in the risk of PE and IUGR in the second pregnancy for unit increase in interpregnancy BMI by BMI categories in the first pregnancy. **c**, **d** Change in the risk of PE and IUGR in the second pregnancy for unit decrease in interpregnancy BMI by BMI categories in the first pregnancy.

The risk of PE and IUGR in the second pregnancy for each 1 kg/m^2^ increase or decrease in interpregnancy BMI is depicted in Fig. 2. Overall, as well as in each BMI category at the time of the first pregnancy, the risk of PE increased while the chance of IUGR reduced with every unit increase in interpregnancy BMI (Fig. 2a and Fig. 2b). Women in the underweight category at the start of the first pregnancy were found to be the most sensitive to this change and possessed a 16% increased risk of PE and an 11% reduction in the risk of IUGR per 1 kg/m^2^ increase in interpregnancy BMI. In contrast, a 1 kg/m^2^ increase in interpregnancy BMI for women in the obesity category only increased their risk of PE, or reduced their risk of IUGR, by 6%. Similarly, reducing weight between pregnancies was associated with a very large increase in the risk of IUGR in underweight women but weight reduction in women with obesity had little impact on their risk of PE (Fig. 2c and Fig. 2d).

The association observed per 1 kg/m^2^ interpregnancy weight change and high birthweight (>4000 g) is also highlighted in Supplementary Figure 1. The risk of a birthweight >4000 g increased with every 1 kg/m^2^ increase in interpregnancy BMI except for women with obesity at the start of their first pregnancy where the risk remained unchanged (Supplementary Figure 1). Conversely, a 1 kg/m^2^ decrease in interpregnancy BMI reduced the risk of high birthweight across all BMI categories (Supplementary Figure 1).

## Discussion

### Main findings

An analysis of pooled data from three European countries confirmed that an interpregnancy BMI increase of ≥2 kg/m^2^ between the first two pregnancies increased the risk of PE and high birth weights but did not markedly affect the risk of any other complications in the second pregnancy. In comparison, a reduction in BMI of ≥2 kg/m^2^ increased the risk of IUGR and preterm birth but reduced the risk of instrumental delivery and high birth weights. In addition, each unit increase in BMI was associated with an increase in the risk of PE and high birthweight but a reduction in the risk of IUGR. On the other hand, each 1 kg/m^2^ reduction, increased the risk of IUGR while decreasing the risk of high birth weights. Women who were underweight at the start of their first pregnancy were most sensitive to interpregnancy BMI change. Previous pregnancy complications (PE or IUGR in the first pregnancy) were the strongest predictor of the same complication occurring in the next pregnancy irrespective of the interpregnancy change in BMI.

### Strengths and limitations

To our knowledge, this is the first study that investigated individualised evidence on perinatal outcomes in relation to BMI change between first and second pregnancies. The main strength of our study is the use of population-based data from three countries which provided a large representative sample for covariate-adjusted stratified analyses as well as an increased power of generalisability. In addition, further key strengths of this study included robust study design, accurate data collection, and statistical analysis. That is, all BMI data included were obtained from accurate weight and height measurements taken at the first antenatal visit by trained professionals which negated any recall, reporting, and potential PE-induced fluid retention bias. The main limitation of our results stem from the lack of standardisation in the definition of variables across the three registries. For example, in the Finnish registry, social class/socioeconomic position was recorded as white/blue-collar workers according to the mother’s occupation during pregnancy while the Maltese database used maternal education, and the AMND by the Registrar general’s classification based on the father’s occupation. These disparate variables were homogenised by categorising each into a binary variable (deprived/not deprived) however, this may still have lost some subtle nuances in the process. Due to missing or improbable data, we were unable to adjust for gestational weight gain, blood pressure, and medication such as aspirin in the second pregnancy. In addition, total gestational weight gain in the first pregnancy and pre-existing maternal diseases such as kidney disease, or chronic hypertension were unavailable. Moreover, gestational diabetes mellitus (GDM) and hypertension in pregnancy were not included due to accuracy concerns within our datasets. For instance, some of our data pre-dates the agreed scientific definition of GDM and due to the coding criteria for the datasets being hierarchical such that pregnancy hypertension and proteinuria is classified as PE and PE and pregnancy hypertension are mutually exclusive conditions, both GDM and pregnancy hypertension could not be included. Furthermore, although access and management of care during pregnancy are standardised in all three countries, population differences are likely to still remain. It should also be noted that the biggest contribution of data came from the Finnish registry, and therefore may have overwhelmed the smaller datasets from the two other countries. However, to circumvent this, we accounted for the country of origin as a covariate in the model. To mitigate any change in clinical practice or trend in the prevalence of overweight and pregnant women who have obesity, the year of birth was included in the adjusted model.

### Context of previous research

A search of the published literature identified three recent systematic reviews on interpregnancy weight change and the risk of perinatal outcomes [32–34]. With all three reporting concordant findings based on similar primary studies [32–34], we will focus on the largest and most robust of these. In their systematic review with meta-analysis and meta-regression, Timmerman *et al*. [32] included > 1 million pregnancies from 30 studies and reported similar findings to the results presented here. Of note, a BMI increase of 1 kg/m^2^ was associated with an increased risk of gestational diabetes and hypertension while only increases of > 3 kg/m^2^ were associated with an increased risk of PE [32]. Moreover, apart from large for date babies, weight loss did not appear to confer any risk reduction for any other complications [32]. Within the same study, the authors found women with a BMI < 25 kg/m^2^, classified as ‘normal weight’ or low-risk women, to be at increased likelihood of subsequent pregnancy complications if they gained weight before the start of the pregnancy [32]. In smaller systematic reviews, Oteng-Ntim et al. and Teulings et al. also report remarkably similar findings [33, 34].

### Meaning of the study

In the age of stratified medicine, this study provides clear evidence to support weight change advice for specific patient subgroups. Women who are underweight in their first pregnancy should be advised to gain weight as even a modest increase is associated with a reduction in the risk of IUGR. Admittedly, this is also associated with an increased risk of PE but given the low background risk of this condition within this patient group, the benefits outweigh the risks. Weight reduction in overweight and women with obesity however is not associated with similar benefits. These women would need to lose an extraordinary amount of weight to achieve the same reduction of PE risk. Similarly, BMI change in women with a history of PE or IUGR appears to generate marginal effects on their risk of complication recurrence and should perhaps be managed with close monitoring and aspirin administration [35]. In a recent study, metformin has shown initial promise in preventing early-onset PE in high-risk women and is perhaps also worth considering as a management option [36].

### Clinical implications

Currently, NICE (National Institute of Clinical Excellence) guidelines suggest that women with a BMI higher than 30 kg/m^2^ at their postnatal check-up are referred for weight loss advice [35]. However, the results presented here demonstrate that a previous history of complication, and moderate changes in BMI between the first and second pregnancy, can lead to an altered risk of adverse pregnancy outcomes such as PE and IUGR. This indicates that advice regarding weight loss or weight gain should be tailored to individual women based on their early pregnancy BMI category and the background risk of pregnancy complications such as PE or IUGR. For example, a woman in the underweight BMI category at the time of the first pregnancy should be advised to gain weight for the second pregnancy as this would be beneficial in reducing her risk of having an IUGR baby. Although weight gain in this category of women may increase their risk of developing PE, the risk in this patient subgroup has been found to be of minor concern [24]. The reverse is true for weight loss and IUGR, where the risk is known to be high [19, 24]. As highlighted in a relatively recent systematic review and meta-analysis, there is a significant deficit of relevant and high-quality studies investigating interpregnancy weight change and adverse pregnancy outcomes including PE risk [37]. Therefore, the results presented here will help to improve the understanding of the impact of interpregnancy weight change on the risk of PE and IUGR and will help provide evidence-based weight guidance to enable better risk stratification of women.

## Conclusion

In this cohort study using a combined data set from three different countries, we found an increased risk of PE and high birthweight (>4000 g) with an interpregnancy BMI increase of ≥2 kg/m^2^. However, an increased BMI between the first and second pregnancies reduced the rate of IUGR. Other than reducing the risk of a high birthweight and instrumental delivery, weight loss between pregnancies did not confer any reduction in the risk of adverse perinatal outcomes but increased the risk of IUGR and preterm delivery. There was very little benefit of weight loss seen in high-risk groups such as women with obesity or women with a previous history of pregnancy complications. Women who were in the underweight category at the start of their first pregnancy were most sensitive to interpregnancy BMI change.

Ultimately, interpregnancy weight change has a modest influence on the risk of the most clinically significant outcomes, PE and IUGR, in differing directions. Prior history of these conditions has the strongest influence on the likelihood of occurrence in the second pregnancy. Therefore, interpregnancy weight change advice between the first and second pregnancy should be tailored to each patient based on their BMI and previous pregnancy history to help benefit maternal and offspring health.

## Supplementary information

Supplementary Figure 1

## References

[1] Kim SS, Zhu Y, Grantz KL, Hinkle SN, Chen Z, Wallace ME (2016). Obstetric and neonatal risks among obese women without chronic disease. Obstet Gynecol.

[2] Scott-Pillai R, Spence D, Cardwell CR, Hunter A, Holmes VA (2013). The impact of body mass index on maternal and neonatal outcomes: a retrospective study in a UK obstetric population, 2004-2011. BJOG..

[3] Ovesen P, Rasmussen S, Kesmodel U (2011). Effect of prepregnancy maternal overweight and obesity on pregnancy outcome. Obstet Gynecol.

[4] Schummers L, Hutcheon JA, Bodnar LM, Lieberman E, Himes KP (2015). Risk of adverse pregnancy outcomes by prepregnancy body mass index: a population-based study to inform prepregnancy weight loss counseling. Obstet and Gynecol.

[5] Lisonkova S, Muraca GM, Potts J, Liauw J, Chan WS, Skoll A (2017). Association between prepregnancy body mass index and severe maternal morbidity. J Am Med Assoc.

[6] Hanson M, Barker M, Dodd JM, Kumanyika S, Norris S, Steegers E (2017). Interventions to prevent maternal obesity before conception, during pregnancy, and post partum. Lancet Diabetes Endocrinol.

[7] Catalano P, Demouzon SH (2015). Maternal obesity and metabolic risk to the offspring: Why lifestyle interventions may have not achieved the desired outcomes. Int J Obes.

[8] Bogaerts A, Van Den Bergh BRH, Ameye L, Witters I, Martens E, Timmerman D (2013). Interpregnancy weight change and risk for adverse perinatal outcome. Obstet Gynecol.

[9] Bender W, Hirshberg A, Levine LD (2019). Interpregnancy body mass index changes: distribution and impact on adverse pregnancy outcomes in the subsequent pregnancy. Am J Perinatol.

[10] Benjamin RH, Littlejohn S, Canfield MA, Ethen MK, Hua F, Mitchell LE (2019). Interpregnancy change in body mass index and infant outcomes in Texas: a population-based study. BMC Pregnancy Childbirth.

[11] Getahun D, Ananth CV, Peltier MR, Salihu HM, Scorza WE (2007). Changes in prepregnancy body mass index between the first and second pregnancies and risk of large-for-gestational-age birth. Am J Obstet Gynecol.

[12] Glazer NL, Hendrickson AF, Schellenbaum GD, Mueller BA (2004). Weight change and the risk of gestational diabetes in obese women. Epidemiology..

[13] Hoff GL, Cai J, Okah FA, Dew PC (2009). Pre-pregnancy overweight status between successive pregnancies and pregnancy outcomes. J Women’s Heal.

[14] Lynes C, McLain AC, Yeung EH, Albert P, Liu J, Boghossian NS (2017). Interpregnancy weight change and adverse maternal outcomes: a retrospective cohort study. Ann Epidemiol.

[15] Sorbye LM, Skjaerven R, Klungsoyr K, Morken NH (2017). Gestational diabetes mellitus and interpregnancy weight change: a population-based cohort study. PLoS Med.

[16] Ziauddeen N, Wilding S, Roderick PJ, Macklon NS, Alwan NA (2019). Is maternal weight gain between pregnancies associated with risk of large-for-gestational age birth? Analysis of a UK population-based cohort. BMJ Open.

[17] Sorbye L, Cnattingius S, Skjaerven R, Klungsoyr K, Wikström A, Kvalvik L (2020). Interpregnancy weight change and recurrence of gestational diabetes mellitus: a population‐based cohort study. BJOG..

[18] Tabet M, Banna S, Luong L, Kirby R, Chang JJ. Pregnancy outcomes after preeclampsia: the effects of interpregnancy weight change. Am J Perinatol. 2020; 10.1055/s-0040-1713000. 10.1055/s-0040-1713000 32521560

[19] Wallace JM, Bhattacharya S, Campbell DM, Horgan GW (2016). Inter-pregnancy weight change and the risk of recurrent pregnancy complications. PLoS One.

[20] Wellings K, Jones KG, Mercer CH, Tanton C, Clifton S, Datta J (2013). The prevalence of unplanned pregnancy and associated factors in Britain: findings from the third National Survey of Sexual Attitudes and Lifestyles (Natsal-3). Lancet..

[21] Villamor E, Cnattingius S (2006). Interpregnancy weight change and risk of adverse pregnancy outcomes: a population-based study. Lancet..

[22] Whiteman VE, Rao K, Duan J, Alio A, Marty PJ, Salihu HM (2011). Changes in prepregnancy body mass index between pregnancies and risk of preterm phenotypes. Am J Perinatol.

[23] Getahun D, Ananth CV, Oyelese Y, Chavez MR, Kirby RS, Smulian JC (2007). Primary preeclampsia in the second pregnancy: effects of changes in prepregnancy body mass index between pregnancies. Obstet Gynecol.

[24] Wallace JM, Bhattacharya S, Campbell DM, Horgan GW (2014). Inter-pregnancy weight change impacts placental weight and is associated with the risk of adverse pregnancy outcomes in the second pregnancy. BMC Pregnancy Childbirth.

[25] Getahun D, Kaminsky LM, Elsasser DA, Kirby RS, Ananth CV, Vintzileos AM (2007). Changes in prepregnancy body mass index between pregnancies and risk of primary cesarean delivery. Am J Obstet Gynecol.

[26] Whiteman VE, Aliyu MH, August EM, McIntosh C, Duan J, Alio AP (2011). Changes in prepregnancy body mass index between pregnancies and risk of gestational and type 2 diabetes. Arch Gynecol Obstet.

[27] Whiteman VE, Crisan L, McIntosh C, Alio AP, Duan J, Marty PJ (2011). Interpregnancy body mass index changes and risk of stillbirth. Gynecol Obstet Invest.

[28] Whiteman VE, McIntosh C, Rao K, Mbah AK, Salihu HM, Interpregnancy BMI (2011). change and risk of primary caesarean delivery. J Obstet Gynaecol (Lahore).

[29] Ayorinde AA, Wilde K, Lemon J, Campbell D, Bhattacharya S (2016). Data Resource Profile: the aberdeen maternity and neonatal databank (AMND). Int J Epidemiol.

[30] Medical Birth Register - THL. https://thl.fi/en/web/thlfi-en/statistics/information-on-statistics/register-descriptions/newborns (Accessed 6 March 2021).

[31] Mallia MM, Scicluna MC, Mercieca ME National Obstetric Information System (NOIS) Malta. https://health.gov.mt/en/dhir/Pages/Registries/births.aspx (Accessed 6 March 2021).

[32] Timmermans YEG, Kant KDG, Oosterman EO, Spaanderman MEA, Villamor‐Martinez E, Kleijnen J (2020). The impact of interpregnancy weight change on perinatal outcomes in women and their children: A systematic review and meta‐analysis. Obes Rev.

[33] Oteng-Ntim E, Mononen S, Sawicki O, Seed PT, Bick D, Poston L (2018). Interpregnancy weight change and adverse pregnancy outcomes: a systematic review and meta-analysis. BMJ Open.

[34] Teulings NEWD, Masconi KL, Ozanne SE, Aiken CE, Wood AM (2019). Effect of interpregnancy weight change on perinatal outcomes: systematic review and meta-analysis. BMC Pregnancy Childbirth.

[35] Recommendations | Weight management before, during and after pregnancy | Guidance | NICE. https://www.nice.org.uk/guidance/ph27 (Accessed 6 March 2021).

[36] Cluver C, Walker SP, Mol BW, Hall D, Hiscock R, Brownfoot FC (2019). A double blind, randomised, placebo-controlled trial to evaluate the efficacy of metformin to treat preterm pre-eclampsia (PI2 Trial): Study protocol. BMJ Open.

[37] Teulings NEWD, Masconi KL, Ozanne SE, Aiken CE, Wood AM (2019). Effect of interpregnancy weight change on perinatal outcomes: systematic review and meta-analysis. BMC Pregnancy Childbirth.

