# Supplementary figures and images for: Does interpregnancy BMI change affect the risk of complications in the second pregnancy? Analysis of pooled data from Aberdeen, Finland and Malta
Source: Int J Obes (Lond). 2021 Oct 4;46(1):178–85. doi: 10.1038/s41366-021-00971-7 (PMC8748194; doi:10.1038/s41366-021-00971-7)

**a**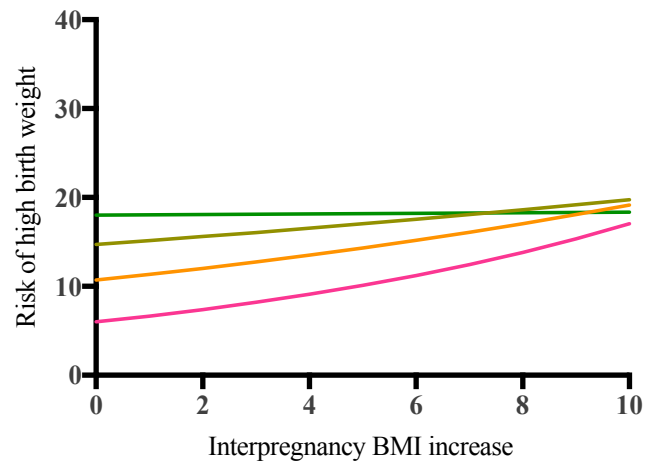**b**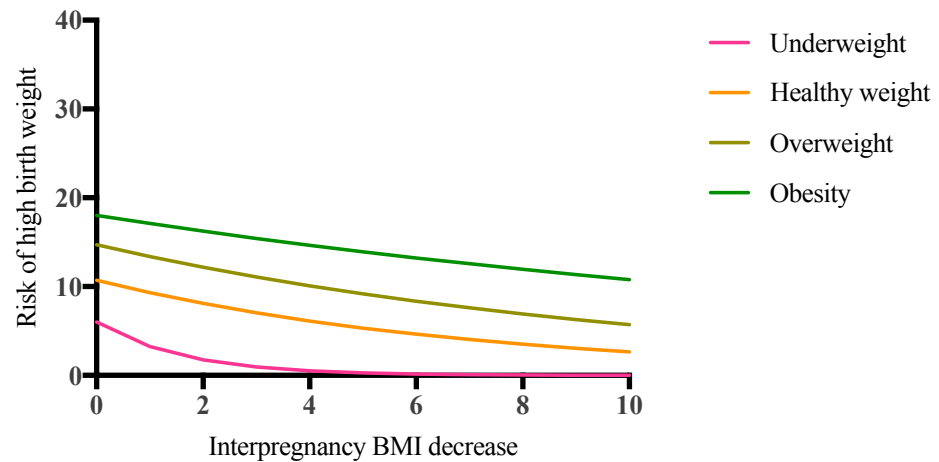

Supplement: Supplementary file 1 — Supplementary Figure 1 [file 41366_2021_971_MOESM1_ESM.pdf]
